# BRCA1 affects the resistance and stemness of SKOV3‐derived ovarian cancer stem cells by regulating autophagy

**DOI:** 10.1002/cam4.1975

**Published:** 2019-01-12

**Authors:** Yue You, Fang‐Fang Bi, Ying Jiang, Ye‐Tao Xu, Yuan‐Yuan An, Da Li, Qing Yang

**Affiliations:** ^1^ Department of Obstetrics and Gynecology Shengjing Hospital of China Medical University Shenyang China; ^2^ Department of Obstetrics, Gynecology, and Reproductive Sciences Yale School of Medicine New Haven Connecticut

**Keywords:** autophagy, BRCA1, cisplatin, drug resistance, ovarian cancer stem cells

## Abstract

Breast cancer 1 (BRCA1) and autophagy both play a significant role in drug resistance. However, little is known about the dynamic cross talk between BRCA1 and autophagy in the regulation of drug sensitivity. Here, we investigated the drug resistance‐associated regulation of BRCA1 in epithelial ovarian cancer stem cells (EOCSCs). The results indicated that BRCA1 could regulate drug resistance in EOCSCs. Autophagy played a significant role in the stemness maintenance and was a key mechanism underlying the survival against chemotherapy in EOCSCs. Further investigation found that BRCA1 could regulate drug resistance of EOCSCs through autophagy. Meanwhile, changes in the level of autophagy provided feedback regarding the expression of BRCA1. Inhibition of autophagy activity could effectively reduce the resistance of EOCSCs caused by BRCA1. In addition, BRCA1 was able to regulate cellular apoptosis and cell cycle progression under the action of cisplatin through autophagy, indirectly affecting the drug sensitivity of EOCSCs. The present results highlight a novel relationship between BRCA1 and autophagy, which may provide insight into the etiology of BRCA1‐associated ovarian cancer, and improve our understanding of resistance mechanisms in ovarian cancer.

## 1 INTRODUCTION

Ovarian cancer mortality ranks first among gynecological malignancies. According to the statistics of the American Cancer Society, an estimated 22 240 new cases and 14 070 deaths from ovarian cancer are predicted in 2018.^1^ Cytoreduction is the first‐line treatment for ovarian cancer in combination with other adjuvant treatments; however, patients with ovarian cancer often develop drug resistance, in addition to showing a high risk of postoperative recurrence.^2^, ^3^, ^4^ The high rate of cancer recurrence is attributed to cancer stem cells according to recent studies. Scientists first isolated and identified epithelial ovarian cancer stem cells (EOCSCs) from ascites of patients with advanced ovarian serous adenocarcinoma in 2005.^5^ EOCSCs are associated with chemoresistance in ovarian cancer because of their self‐renewal capacity and multiple differentiation potential.

Breast cancer 1 (BRCA1) is a tumor suppressor gene. Nearly 20% of cases of high‐grade ovarian serous adenocarcinoma have BRCA germline mutations, of which more than 40% do not have a family history of cancer.^6^ BRCA1 plays an important role in many life processes, including transcriptional regulation, double‐stranded DNA damage repair, transcription‐coupled DNA repair, cell cycle regulation, gene silencing, and energy metabolism regulation.^7^ Our previous studies confirmed that BRCA1 is a key factor in the recurrence and drug resistance of ovarian cancer.^8^ Emerging evidence suggests that the relationship between autophagy and cancers is twofold. Autophagy inhibits inflammation and promotes genomic stability, while also protecting cancer cells from stress‐related damage.^9^, ^10^

Despite data confirming the involvement of BRCA1 in the regulation of drug resistance, the mechanism underlying the BRCA1‐mediated resistance of ovarian cancer cells remains uncertain. In the present study, we constructed a unique in vitro ovarian cancer model using a serum‐free sphere suspension culture to study the process of drug resistance. The differential expression of BRCA1 in EOCSCs was evaluated, as well as the relationship between autophagy and the resistance of ovarian cancer to cisplatin‐based chemotherapy. Our findings demonstrated that BRCA1 mediated ovarian cancer characteristics including drug resistance and stemness of EOCSCs by modulating autophagy. Autophagy was identified as a key factor in the maintenance of self‐renewal and drug resistance in EOCSCs and may be a potential therapeutic target for refractory ovarian cancer.

## 2 MATERIALS AND METHODS

### 2.1 Ethical statement

The investigation was carried out in accordance with the ethical standards of the Helsinki Declaration (2013) of the World Medical Association. All clinical samples in the study were collected with patient consent and used under approval from the Institutional Review Board at China Medical University.

### 2.2 Patients and tissue collection

Serous ovarian cancer patients (10 chemosensitive and 10 chemoresistant) were enrolled between 2014 and 2016. Fresh tumor samples were obtained from the Department of Obstetrics and Gynecology, Shengjing Hospital of China Medical University. Tumor samples were derived from patients undergoing primary surgical resection before receiving chemotherapy or radiotherapy. Samples were stained with hematoxylin and eosin for histopathological diagnosis and grading by three staff pathologists using the World Health Organization criteria.

### 2.3 Cell culture, enrichment, cisplatin treatment, transfection, cell proliferation assay, and cell apoptosis assay

The human ovarian cancer cell line SKOV3 was purchased from Nanjing Kebai Biotechnology Co., Ltd. (Nanjing, China) and maintained in McCoy's 5A medium (Sigma‐Aldrich, St. Louis, MO, USA) with 0.23% NaHCO_3_ and 10% fetal bovine serum (Invitrogen, Carlsbad, CA, USA). EOCSCs enriched from SKOV3 cells were maintained in DMEM/F12 medium (Gibco, Carlsbad, CA, USA) supplemented with 10% knockout serum, 1% sodium pyruvate, 1% GlutaMAX, 1% 2‐mercaptoethanol, 1% MEM NEAA, 0.4% bFGF (Novus, CO, USA), and 0.8% EGF (Novus, CO, USA) on ultra‐low attachment 6‐well plates (Corning, NY, USA). Cis‐diamineplatinum (II) dichloride was purchased from Solarbio (Beijing, China). Rapamycin (Sirolimus), Torkinib (PP242), 3‐Methyladenine (3‐MA), and hydroxychloroquine sulfate (CQ) were purchased from Selleck Chemicals (Houston, TX, USA) and used according to the methods on the official website. Rapamycin: 100 nmol/L, 36 hours; Torkinib: 2.5 μmol/L, 24 hours; 3‐MA: 10 mmol/L, 24 hours; CQ: 100 μmol/L, 48 hours. Lentiviral vectors expressing short hairpin RNAs (shRNAs) against BRCA1 were obtained from Hanbio Biotechnology Co., Ltd. (Shanghai, China). The core knockout sequence is shown in Table S1. A nonsilencing shRNA sequence was used as a negative control. BRCA1‐overexpression plasmids were obtained from Genechem Co., Ltd. (Shanghai, China). After 48 hours of cultivation, the corresponding antibiotic drugs were added into the medium for screening. Small interfering RNAs (siRNAs) against Beclin‐1 and Atg‐5 and negative control siRNAs were obtained from Shanghai GenePharma Biotechnology Co., Ltd. (Shanghai, China). All the synthesized sequences are shown in Table S1. Cisplatin was selected as the apoptosis inducer to simulate chemotherapy. The fifty percent of growth inhibition (IC_50_) of cisplatin for EOCSCs was 47.821 μmol/L, and therefore, EOCSCs were treated with 50 μmol/L cisplatin. After cisplatin treatment of 48 hours, cell proliferation was assessed using the Cell‐Light™ EdU Apollo^®^643 In Vitro Imaging Kit (Ribobio, Guangzhou, China) according to the manufacturer's instructions and imaged with a fluorescence microscope (Nikon, Japan) with original magnification of 20×.

### 2.4 Cell viability assay and growth curve

Approximately 5000 EOCSCs or SKOV3 cells per well were plated in 96‐well plates. After incubation with cisplatin for 48 hours, the medium was replaced and 10% Cell Counting Kit‐8 assay reagent was added (Bimake, Shanghai, China). The plate was incubated for an additional 2 hours. The absorbance at 450 nm was detected using a microplate reader. Growth curves were drawn on days 1, 2, 3, and 4.

### 2.5 Protein preparation, SDS‐PAGE, and Western blot analysis

Total tissue and cell proteins were extracted following the instructions of RIPA lysate (Beyotime, Shanghai, China). Western blot was performed using 40‐50 μg of protein lysates. Proteins were separated using 12.5% SDS‐PAGE and transferred onto PVDF membranes (AP124P, Merck, Germany). The remaining steps were performed strictly in accordance with the instructions of each antibody. Protein bands were detected using enhanced chemiluminescence (32109, Thermo Scientific, Waltham, MA, USA). The antibodies used in the study, including origin and dilution, are listed in Table S2. Glyceraldehyde phosphate dehydrogenase (GAPDH) was used as a reference.

### 2.6 RNA extraction and real‐time quantitative PCR

Total tissue and cell RNAs were isolated using the RNAiso Plus reagent (Takara Bio, Dalian, China). Genomic DNA was removed, and approximately 1000 ng RNA was reverse transcribed to cDNA using the PrimeScript™ RT reagent Kit with gDNA Eraser (Perfect Real Time) (Takara Bio, Dalian, China). Real‐time qPCR was performed using cDNA and the appropriate primers with SYBR^®^ Premix Ex Taq™ II (Tli RNaseH Plus) (Takara Bio, Dalian, China). All kits were used according to the manufacturer's instructions. The primer sequences are shown in Table S3. The PCR reactions were performed on ABI 7500 Fast (Life Technologies, Carlsbad, CA, USA) in triplicate and validated by the presence of one single peak in the melt curve. Gene expression was calculated relative to that of GAPDH using the 2^−△△Ct^ method.

### 2.7 Flow cytometry analysis

The expression of the cell surface molecule was determined by staining cells with CD44‐APC, human (clone: DB105) and CD133/2(293C3)‐APC, human (clone: 293C3) (Miltenyi Biotec GmbH, Germany) according to the instruction book. Mouse IgG1‐APC and Mouse IgG2b‐APC (Miltenyi Biotec GmbH, Germany) were used as isotype controls. Cellular apoptosis was detected by flow cytometry using the Annexin V FITC Apoptosis Detection Kit (DOJINDO, Japan) according to the manufacturer's instructions. In addition, apoptosis was assessed by microscopic imaging (Nikon, Japan) with original magnification of 20×. Cell cycle progression was detected by flow cytometry using the Cell Cycle and Apoptosis Analysis Kit (Beyotime, Shanghai, China). Data were acquired and analyzed using FACSCalibur system (BD Bioscience, Piscataway, NJ, USA).

### 2.8 Immunofluorescence analysis

EOCSCs in good condition were seeded into 6‐well plates at a density of 1 × 10^5^ cells/ml. When the cell confluency reached 50%‐70%, EOCSCs were transfected with mRFP‐GFP‐LC3 autophagy double‐labelled adenovirus for 24 hours using a transfection method specific for suspension cells. Microscope imaging was performed after 48 hours. The yellow spots detected by red‐green fluorescence merging were autophagosomes, the red spots were autophagic lysosomes, and the intensity of the autophagic flow was determined by detecting the expression level of different color spots. Cells were imaged using a fluorescence microscope (Nikon, Japan) with original magnification of 20×.

### 2.9 Statistical analysis

Data were presented as the mean ± standard error (SE) from at least three independent experiments. All statistical analyses were performed with GraphPad Prism 5.0 and IBM Statistics SPSS 22.0. Student's *t* test was performed to evaluate the statistical differences between two groups. One‐way analysis of variance (ANOVA) followed by Scheffe's multiple group comparison was performed to evaluate the statistical differences between different groups. The correlation between the expression levels of two genes was tested by the Pearson correlation coefficient. *P *< 0.05 was considered statistically significant.

## 3 RESULTS

### 3.1 Ovarian cancer‐resistant tissues express high levels of BRCA1 and autophagy

**Figure 1:**
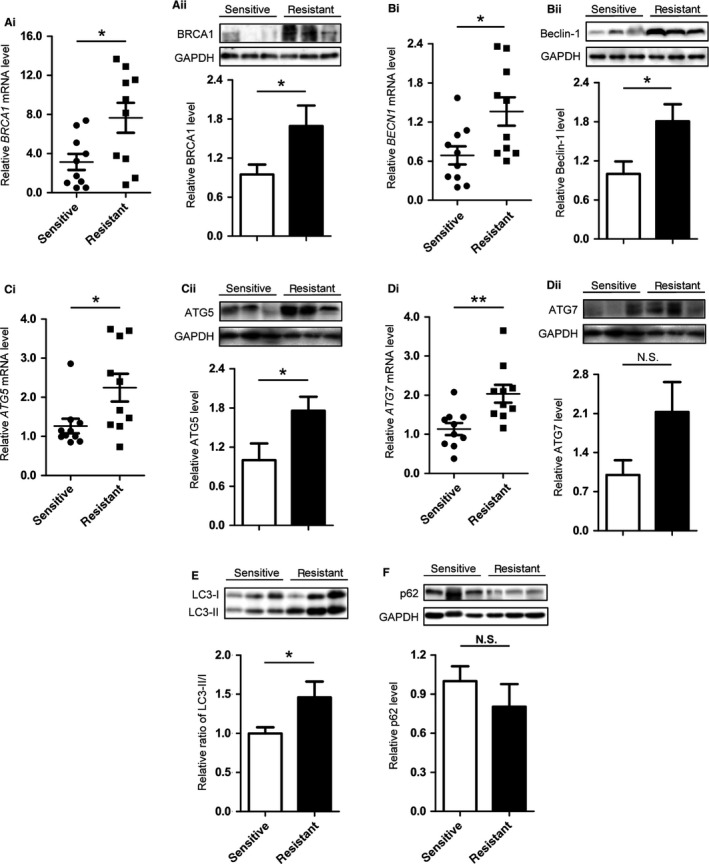
Differences in the expression patterns of BRCA1, Beclin‐1, ATG5, ATG7, p62, and LC3 in differential cisplatin‐sensitive cancers. A, Quantitative data and representative Western blot results of BRCA1 in ovarian cancer tissues with different sensitivity to chemotherapy. B, Quantitative data and representative Western blot results of Beclin‐1 in ovarian cancer tissues with different sensitivity to chemotherapy. C, Quantitative data and representative Western blot results of ATG5 in ovarian cancer tissues with different sensitivity to chemotherapy. D, Quantitative data and representative Western blot results of ATG7 in ovarian cancer tissues with different sensitivity to chemotherapy. E, Representative Western blot results and quantification data of LC3 in ovarian cancer tissues with different sensitivity to chemotherapy. F, Representative Western blot results and quantification data of p62 in ovarian cancer tissues with different sensitivity to chemotherapy. Each spot in the scatter plot represents the relative expression of one independent sample. Glyceraldehyde phosphate dehydrogenase (GAPDH) was used as a control to normalize band density; columns represent mean ± SE. *P < 0.05; **P < 0.01; NS not statistically significant

To investigate the mechanism underlying the drug resistance of ovarian cancer, the expression levels of BRCA1 were detected in platinum‐resistant and platinum‐sensitive ovarian cancer tissues. The results showed that the mRNA and protein levels of BRCA1 were higher in resistant tissues than in sensitive tissues (Figure 1A). The mRNA levels of *BECN1*, *ATG5*, and *ATG7* were higher in resistant tissues than in sensitive tissues (Figure 1B‐i,C‐i,D‐i). The mRNA levels of *ABCG2* and *ABCB1* were also higher in resistant samples (Figure S1A,B). We analyzed the relationship between resistant levels and *BRCA1* levels in ovarian cancer tissue samples. The results showed that *BRCA1* was positively correlated with drug resistance (Figure S1C,D). These results indicated that BRCA1 and autophagy may be involved in the development and maintenance of drug resistance in ovarian cancer. The results of Western blot analysis showed that Beclin‐1, ATG5, and LC3 were markedly higher in resistant tissues than in sensitive tissues (Figure 1B‐ii,C‐ii,E), with a slightly increasing trend in the expression of ATG7 and a slightly decreasing trend in the expression of the autophagy substrate p62 (Figure 1D‐ii,F). Other than these, we detected the protein levels of POU5F1 and NANOG in sensitive and resistant tissues. The results showed that the expression of stem markers in resistant tissues was significantly higher than that in sensitive tissues (Figure S1E,F). The clinicopathologic parameter information of the serous ovarian cancer cases was shown in Table S4.

### 3.2 EOCSCs exhibit higher stemness and drug resistance

**Figure 2:**
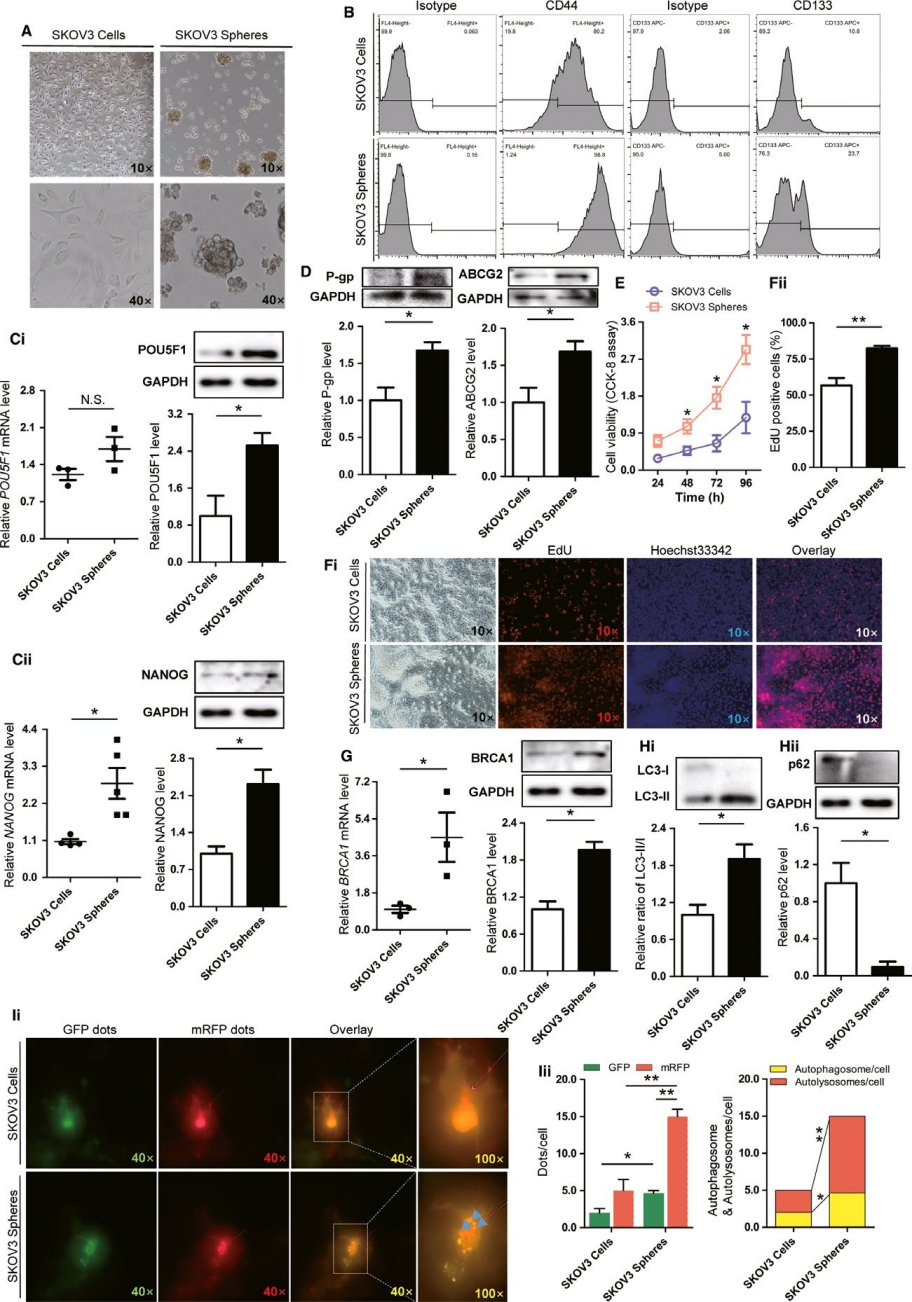
Identification of epithelial ovarian cancer stem cells (EOCSCs) from SKOV3 cells and differences in expression patterns of BRCA1 and autophagy levels. A, Morphology of EOCSCs isolated from SKOV3 cells after 7 d. B, Pluripotency markers CD44 and CD133 measured in SKOV3 cells and EOCSCs. C, Quantitative data and representative Western blot results of POU5F1 and NANOG in SKOV3 cells and EOCSCs. D, Representative Western blot results and quantification data of P‐gp and ABCG2 in SKOV3 cells and EOCSCs. E, Evaluation of the viability of SKOV3 and EOCSCs via CCK‐8 assay. Data represent the average of three independent experiments. F, EdU marker showing the proliferation of SKOV3 and EOCSCs. Red, EdU marked nuclei of proliferative cells; blue, Hoechst 33342 marked nuclei; proliferative activity reflected by the ratio of EdU positive cells to Hoechst 33342 positive cells. G, Quantitative data and representative Western blot results of BRCA1 in SKOV3 cells and EOCSCs. H‐i, Representative Western blot results and quantification data of LC3 in SKOV3 cells and EOCSCs. H‐ii, Representative Western blot results and quantification data of p62 in SKOV3 cells and EOCSCs. I, Autophagy double‐labelled adenovirus (mRFP‐GFP‐LC3) reflecting the autophagy intensity of SKOV3 cells and EOCSCs. The expression of GFP and mRFP in mRFP‐GFP‐LC3 tandem fluorescent protein adenovirus was used to label and trace LC3, and the decrease of GFP could indicate the fusion of lysosome and autophagosome to form autolysosome. Columns represent the mean ± SE of triplicate samples with 20 cells analyzed per sample. Each spot in the scatter plot represents the relative expression of one independent sample. Glyceraldehyde phosphate dehydrogenase (GAPDH) was used as a control to normalize band density; columns represent mean ± SE. *P < 0.05; **P < 0.01; NS not statistically significant

Several methods for extracting cancer stem cells have been reported in different gynecological tumors.^11^, ^12^, ^13^, ^14^, ^15^ Serum‐free suspension culture is widely used to isolate many types of cancer stem cells because it is non‐toxic and effective. SKOV3 is recognized as a commonly used tool for extracting stem cells, due to its high resistance and high expression of stemness markers. SKOV3 cells in the appropriate growth phase were plated in ultra‐low absorbance 6‐well plates at a density of 10^5^ cells per well. After a 7‐day balling cycle (Figure 2A), spherical cells were isolated, harvested by centrifugation, and digested with 0.25% trypsin into single cells for identification. Flow cytometry showed that CD44+ cells accounted for a significant increase in the total number of cells compared with before separation, from 80.2% ± 5.40% to 98.8% ± 3.14%, whereas CD133+ cells increased from 10.8% ± 0.50% to 23.7% ± 5.46% (Figure 2B). The results of real‐time qPCR showed an increasing trend in *NANOG*, *CD44*, and *ABCG2* mRNA levels in EOCSCs compared with those in adherent cells (Figures 2C‐ii and S2A,B). Western blot analysis showed that the expression levels of POU5F1, NANOG, P‐glycoprotein (P‐gp), and ABCG2 were significantly increased in EOCSCs (Figure 2C,D). The results of CCK‐8 showed no significant difference in the number of cells between SKOV3 cells and EOCSCs at 24 hours. However, EOCSCs divided more rapidly than SKOV3 cells between 48 hours and 96 hours (Figure 2E). The EdU cell proliferation assay confirmed that the rate of EdU positive cells was significantly greater in the EOCSCs group than in SKOV3 cells under the action of cisplatin (Figure 2F).

### 3.3 EOCSCs display higher levels of BRCA1 and basal autophagy than SKOV3 cells

To identify the factors responsible for the different biological effects between EOCSCs and parental cells, real‐time PCR and Western blot analysis were performed. The results showed that BRCA1 was upregulated in EOCSCs (Figure 2G). Active autophagy was observed in EOCSCs. The LC3‐II/I ratio was increased and p62 was significantly downregulated in EOCSCs compared with SKOV3 cells (Figure 2H). Consistent with the results of Figure 2H, autophagy double‐labelled adenovirus (mRFP‐GFP‐LC3) reflecting the autophagy intensity showed that autophagy flow was progressive in EOCSCs (Figure 2I). These results suggested that EOCSCs possess higher viability and a higher level of basic autophagy than parent cells.

### 3.4 BRCA1 regulates autophagy and stemness in ovarian cancer cells

**Figure 3:**
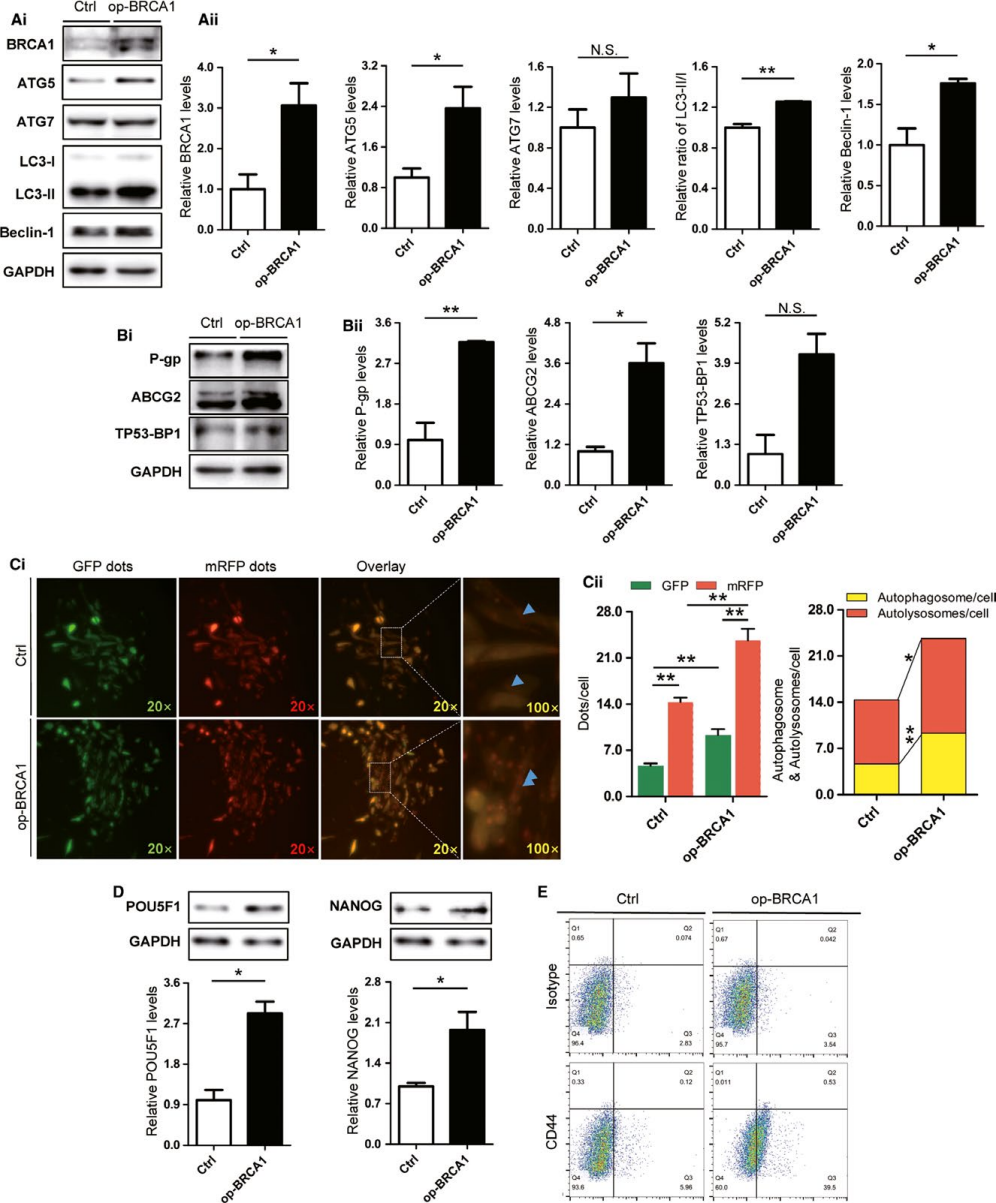
Changes of autophagy and stem markers in ovarian cancer cells after overexpression of BRCA1. A, Cells transfected with BRCA1 overexpression plasmids were selected with kanamycin and verified by Western blot analysis. Representative Western blot results and quantification data of ATG5, ATG7, LC3‐II/I, and Beclin‐1 in EOCSCs transfected with plasmids. B, Representative Western blot results and quantification data of TP53‐BP1, P‐gp, and ABCG2 in EOCSCs transfected with plasmids. C, Autophagy double‐labelled adenovirus (mRFP‐GFP‐LC3) reflecting the autophagy intensity of EOCSCs transfected with plasmids. Columns represent the mean ± SE of triplicate samples with 20 cells analyzed per sample. D, Representative Western blot results and quantification data of POU5F1 and NANOG in EOCSCs transfected with plasmids. E, CD44 measured in differential adherent cells transfected with plasmids. Glyceraldehyde phosphate dehydrogenase (GAPDH) was used as a control to normalize band density; columns represent mean ± SE. *P < 0.05; **P < 0.01; NS not statistically significant

The BRCA1 plasmid was transfected into EOCSCs, and the transfection efficiency was confirmed by Western blot analysis (Figure 3A). Overexpression of BRCA1 upregulated Beclin‐1, ATG5, P‐gp, and ABCG2 (Figure 3A,B). The LC3‐II/I ratio also showed an increasing trend (Figure 3A). The elevated expression of ATG7 and TP53‐BP1 was not statistically significant (Figure 3A,B), although it may have biological significance to some extent. Autophagic flux increased in response to BRCA1 overexpression (Figure 3C), confirming that BRCA1 promoted autophagy in EOCSCs. Western blot results showed that BRCA1 increased the expression of POU5F1 and NANOG (Figure 3D). The differentially adherent cells cultivated in a serum‐free suspension system were collected. The flow cytometry results showed that they possessed a relatively negative CD44 phenotype. However, transfection of the BRCA1 plasmid increased CD44 levels of them (Figure 3E).

### 3.5 Knockdown of BRCA1 and inhibition of autophagy can both reduce the tolerance of EOCSCs to cisplatin reflected in apoptosis increasing and cell cycle arrest under the action of cisplatin

**Figure 4:**
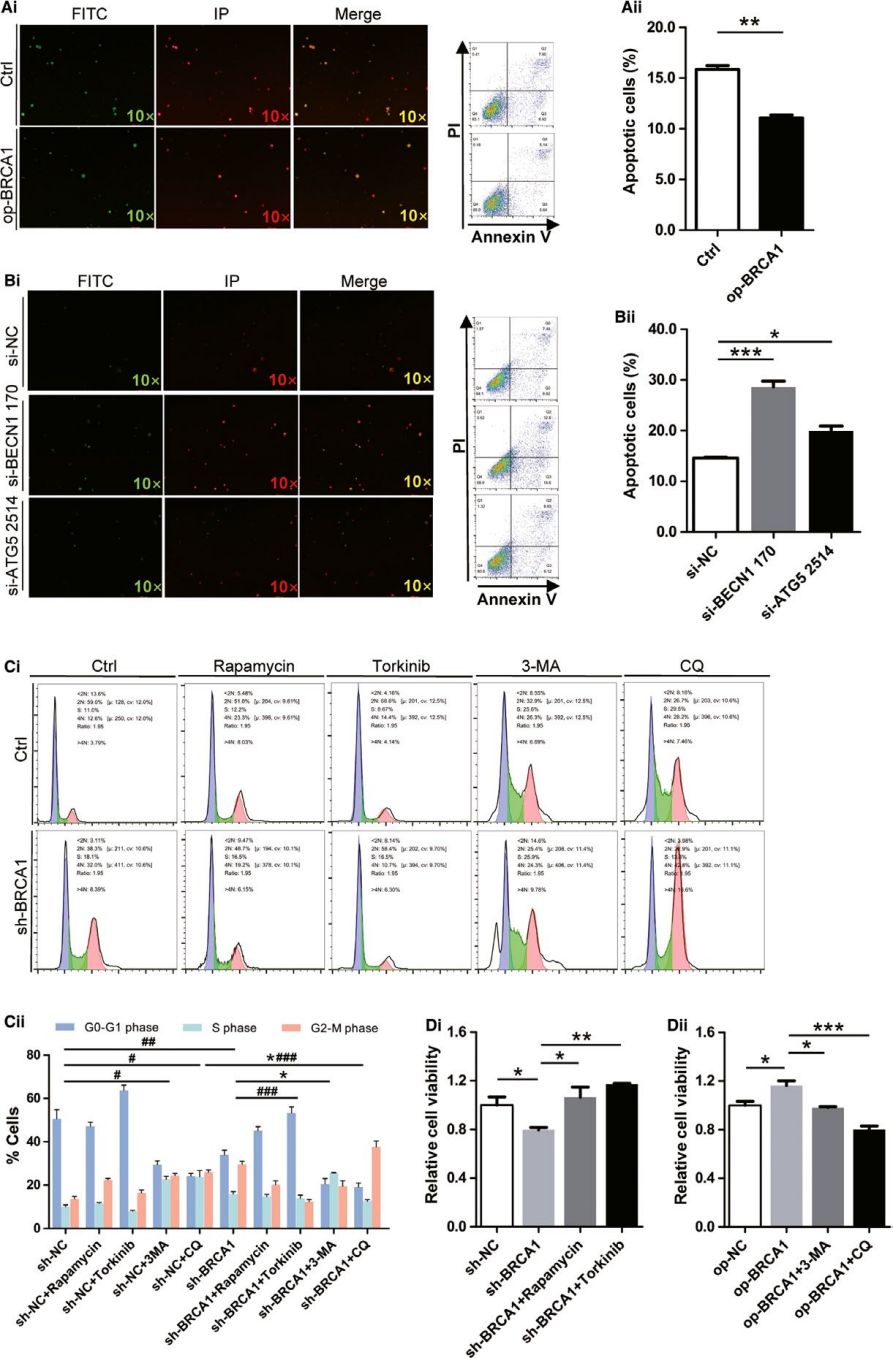
Effects of BRCA1 and autophagy on the apoptosis, cell cycle, and cell viability of EOCSCs. A, Apoptosis levels of EOCSCs transfected with plasmids and detected by flow cytometry and microscopic observation. B, Apoptosis levels of EOCSCs transfected with siRNAs and detected by flow cytometry and microscopic observation. C, BRCA1, autophagy, and dual effects on cell cycle. Columns represent the mean ± SE of triplicate samples; *P < 0.05 in S phase; ^#^ *P* < 0.05, ^##^ *P* < 0.01 in G2/M phase; ^###^ *P* < 0.001 in G2/M phase. In this figure, cells were pretreated with cisplatin (50 μmol/L, 48 h) before detection. D, Evaluation of the viability of EOCSCs after different treatments via CCK‐8 assay. *P < 0.05, **P < 0.01, ***P < 0.001; data represented three independent experiments

To study the chemoresistance mechanism of residual EOCSCs after chemotherapy, cisplatin was used as an apoptosis inducer. The results of apoptosis assays suggested that BRCA1 rescued the apoptosis process in EOCSCs after cisplatin treatment (Figure 4A), which indicated that BRCA1 could enhance the drug resistance of EOCSCs. Pagotto et al reported that autophagy blockade impairs the canonical properties of ovarian cancer stem cells such as self‐renewal/maintenance rather than proliferation inhibition.^16^ In our experiments, EOCSCs were transfected with control siRNA (si‐NC), BECN1 siRNA (si‐BECN1), or ATG5 siRNA (si‐ATG5). Western blot analysis was performed to confirm the transfection efficiency, as shown in Figure S3A,B. Knockdown of both *BECN1* and *ATG5* by siRNA significantly increased cisplatin‐induced cell apoptosis (Figure 4B). A lentivirus knockout vector was used to stably knockdown BRCA1 in EOCSCs. Fluorescence images and Western blot analysis were used to determine the transfection efficiency (Figure S3C). Cisplatin is an alkylating agent; it can negatively regulate cell cycle progression in certain types of tumor cells.^17^, ^18^ In Figure 4C, BRCA1 knockout resulted in a significant G2/M phase arrest in EOCSCs, indicating that the sensitivity of sh‐BRCA1 EOCSCs to cisplatin was significantly increased. BRCA1 silencing affected the process of DNA damage repair, and the DNA damaged by cisplatin caused the replication arrest. Both the defect in replication and DNA damage repair started the cell cycle checkpoint and blocked it in the premitotic phase. This provided sufficient time for DNA damage repair to prevent cells from entering mitosis before they were repaired.^19^ While the EOCSCs were treated with 3‐MA or chloroquine, G2/M phase arrest was observed. Torkinib could effectively alleviate the degree of G2/M phase arrest resulted from sh‐BRCA1. After the cells were treated with 3‐MA, an apoptotic peak appeared. While after treatment with chloroquine, sh‐BRCA1 EOCSCs showed a severe G2/M phase arrest suggesting that DNA was severely damaged and could not be repaired, also, apoptosis was initiated leading to irreversible G2/M phase arrest. 3‐MA can block the formation of autophagosomes. It acts on Vps34 and PI3Kγ to inhibit autophagy.^20^ However, 3‐MA is a selective PI3K inhibitor. The effects of 3‐MA on some biological functions may not only be through inhibiting autophagy. CCK‐8 results showed that knockdown of BRCA1 significantly increased cisplatin‐induced cell death, whereas autophagy alleviated the increase in cisplatin sensitivity caused by BRCA1 deletion to some extent (Figure 4D).

### 3.6 BRCA1 enhances drug resistance and maintains the stemness of EOCSCs via autophagy

**Figure 5:**
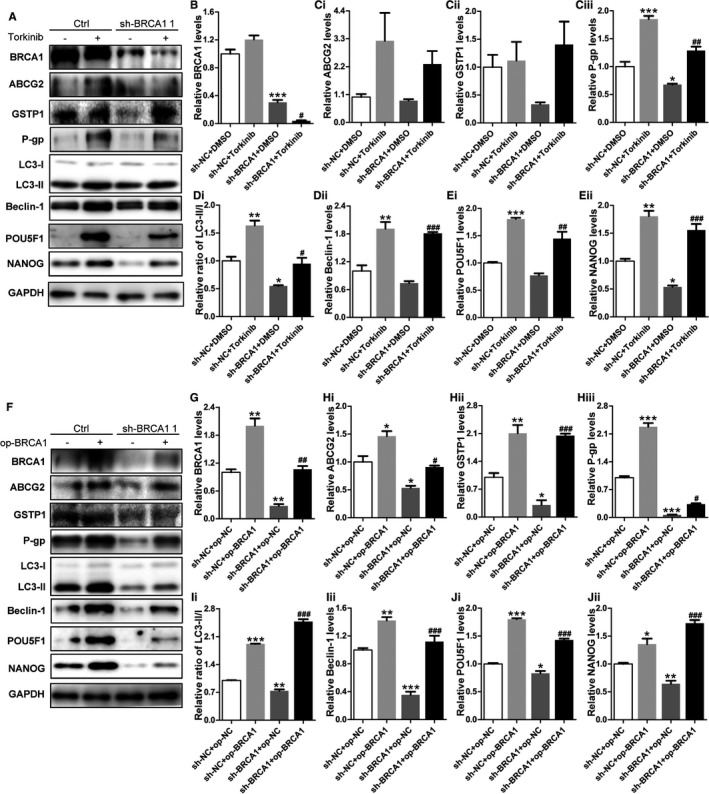
BRCA1 reduces cisplatin sensitivity and enhances stemness in EOCSCs by regulating autophagy. A‐E, Representative Western blot results and quantification data of ABCG2, GSTP1, P‐gp, LC3, Beclin‐1, POU5F1, and NANOG in EOCSCs after different treatments. Glyceraldehyde phosphate dehydrogenase (GAPDH) was used as a control to normalize band density; columns represent the mean ± SE of triplicate samples; *P < 0.05, **P < 0.01, ***P < 0.001 vs the control group; ^#^ *P* < 0.05, ^##^ *P* < 0.01, ^###^ *P* < 0.001 vs the sh‐BRCA1 + DMSO. F‐J, Representative Western blot results and quantification data of ABCG2, GSTP1, P‐gp, LC3, Beclin‐1, POU5F1, and NANOG in EOCSCs after different treatments. GAPDH was used as a control to normalize band density; columns represent the mean ± SE of triplicate samples; *P < 0.05, **P < 0.01, ***P < 0.001 vs the control group; ^#^ *P* < 0.05, ^##^ *P* < 0.01, ^###^ *P* < 0.001 vs the sh‐BRCA1 + op‐NC group.

Autophagy blockade by both pharmacologic and genetic approaches increased the sensitivity of EOCSCs to chemotherapy in vitro. In previous experiments, we found significant changes in drug resistance in groups treated with torkinib, which had similar effect as op‐BRCA1. Knockdown of BRCA1 downregulated NANOG compared with the group transfected with sh‐NC, whereas such a change was not observed in the groups treated with torkinib (Figure 5A). A similar situation was reflected in the expression of P‐gp, a multidrug resistance gene that encodes a permeability glycoprotein. However, the changes of ABCG2 and glutathione S‐transferase pi 1 (GSTP1) were not statistically significant. As shown in Figure 5F, the effect of BRCA1 was rescued by transfection with the BRCA1 overexpression plasmid, and the trend in Beclin‐1 was similar to that in Figure 5A. The expression of BRCA1 was significantly reduced when autophagy activity was enhanced by torkinib (Figure 5A). We can conclude that high expression of BRCA1 is an important factor causing chemotherapy resistance, and it plays a vital role in the maintenance of stemness in EOCSCs. BRCA1 affects cell stemness and drug resistance by regulating autophagy, and the resistance process might be related to P‐gp. Inhibition of autophagic activity might provide a new strategy for overcoming resistance to cisplatin in human ovarian cancer cells.

## 4 DISCUSSION

High mortality, which is a hallmark of ovarian cancer among gynecological tumors worldwide, is related to disease recurrence. Platinum is the first line of chemotherapy for ovarian cancer after surgery. Studies found that approximately 25% of patients showed platinum resistance at the time of the first relapse, and almost all relapsed patients eventually developed platinum resistance.^21^ This indicates that an effective treatment for the primary disease does not produce a response or show efficacy in recurrent disease.

Evidence indicated that BRCA1 plays a critical role in cisplatin resistance.^22^ However, the mechanism underlying platinum resistance in ovarian cancer remains unclear. EOCSCs are associated with drug resistance and disease relapse. Even if tumors rapidly regress at the initial stage of treatment, subsequent use of the same treatment may not be effective.^23^ Rather than rebuilding tumors by differentiation, cancer stem cells are enriched in recurrent diseases and activate self‐renewal pathways that maintain stem‐related characteristics.

We selected the ovarian cancer‐resistant cell line SKOV3 as the parental cell line for the enrichment of stem cells, using previously published culture methods.^24^ Our experimental data indicated that there was a certain correlation between the expression of BRCA1 and the drug resistance genes in ovarian cancer tissues and also demonstrated a novel mechanism of BRCA1‐mediated autophagy‐related regulation of drug resistance. The expression of BRCA1 in ovarian cancer tissues was lower than that in normal tissues; however, high levels of BRCA1 suggest a poor prognosis in ovarian cancer. BRCA1 is an important factor in DNA damage repair, and changes in the DNA repair capacity of damaged cells in tumors are important factors mediating tumor resistance to platinum drugs.^25^ The present results indicated that BRCA1 and autophagy levels were elevated in EOCSCs. Furthermore, the basal level of BRCA1 affected the regulation of autophagy.

Autophagy can promote cell survival instead of causing cell death. It serves as an important mechanism for maintaining genetic integrity when cells are subjected to metabolic stress, drug therapy, and radiation damage. Therefore, inhibition of autophagy in breast, prostate, and colon cancer cells can increase the sensitivity of tumor cells to radiotherapy and chemotherapy.^26^ Autophagy can be regulated at both transcriptional and post‐translational levels.^27^ Previous studies indicated that BRCA1 negatively regulates the expression of EGFR in ovarian cancer.^8^ Tan et al showed that inhibiting EGFR signaling can induce autophagy in tumor cells.^28^ Active EGFR can bind to Beclin‐1, resulting in multiple tyrosine phosphorylation of Beclin‐1 and increased binding with inhibitors, thereby decreasing Beclin‐1‐associated Vps34 kinase activity.^29^ This may be one of the mechanisms by which BRCA1 regulates autophagy in ovarian cancer. In addition, BRCA1 played an important role in the maintenance of stemness in EOCSCs which might be attributed to the role of autophagy in the maintenance of stemness. García‐Prat et al showed, for the first time, that autophagy is essential for the maintenance of the quiescent state in mouse stem cells, and loss of autophagy in senescent cells or genetic damage to autophagy in young cells accelerates cell death, negatively affecting function and quantity.^30^ These authors proposed that autophagy is essential for the function of hematopoietic stem cells, and autophagy inhibition might lead to the loss of stemness and malignancy.^31^ Yang et al reported a positive correlation between LC3, an important autophagy molecule, and the cancer stemness markers ALDH, CD44, and CD133 in pancreatic cancer tissues via microarrays analysis, and their high expression is associated with poor prognosis of patients.^32^ Xue et al discovered in hepatic progenitor cells (HPCs) that the loss of autophagy reduced colony formation and sphere formation abilities. Autophagy defects inhibit the homologous recombination (HR) pathway of DNA damage repair in HPCs. The results showed that autophagy contributes to the maintenance of cell stemness; however, in contrast to García‐Prat's view, Xue supported that autophagy reduces the susceptibility to neoplastic transformation in HPCs.^33^ In the present study, the activation and enhancement of autophagy not only reduced the sensitivity of EOCSCs to cisplatin by upregulating the expression of drug resistance regulatory proteins, but also reduced cell death by activating DNA damage repair, which was consistent with the anti‐apoptotic effect of BRCA1. Autophagy also regulated cell cycle progression and inhibited G2/M phase arrest, consistent with previous findings that autophagy regulates starvation‐induced cell cycle arrest and promotes cell entry into G0/G1 quiescence to counteract stress.^34^ The activation of autophagy reduces the proportion of cells in the fast dividing phase, which also explains why autophagy allows tumor cells to escape the killing by targeted chemotherapy drugs. Li et al proposed that ATG5‐mediated autophagy of proximal tubular epithelial cells is an important host defense mechanism that prevents renal fibrosis by blocking G2/M arrest.^35^ We found that BRCA1 showed a compensatory decrease when autophagy activity was enhanced. There might be a special feedback mechanism for the regulation of autophagy by BRCA1. Therefore, we will systematically study how this feedback regulation is implemented.

The present findings provide insight into the regulatory relationship between BRCA1 and autophagy and enhance our understanding of the molecular mechanisms underlying BRCA1‐associated ovarian cancer cisplatin resistance. However, the pathway by which autophagy regulates and affects drug resistance remains unclear. In future experiments, we will continue to study the important role of BRCA1 in resistance regulation and dig deeper into the mechanisms of autophagy functions in ovarian cancer.

The promotion and maintenance of tumor resistance is a complex network. Inhibiting resistance pathways combined with other chemotherapeutic approaches may lead to new effective strategies preventing cancer relapse and drug resistance. More importantly, personalized treatment plans should be developed for each patient, and further research may identify biological targets for personalized treatment.

## CONFLICT OF INTEREST

The authors have no conflict of interest.

## Supporting information

FigS1

FigS2

FigS3
