# Supplementary material for: BRCA1 affects the resistance and stemness of SKOV3‐derived ovarian cancer stem cells by regulating autophagy
Source: Cancer Med. 2019 Jan 12;8(2):656–68. doi: 10.1002/cam4.1975 (PMC6382722; doi:10.1002/cam4.1975)
Supplement: Supplementary file 4 [file CAM4-8-656-s004.docx]

| Table S1. All the synthesized sequences applied in this study | |
| --- | --- |
| ID | seq |
| BRCA1(NM_007299)-P1 | TACCGACTCAGATCTCGAGCGCCACCATGGATTTATCTGCTCTTCG |
| BRCA1(NM_007299)-P2 | GATCCCGGGCCCGCGGTACCGTCACATCTGCCCAATTGCATGGAAG |
| shRNA NC Top strand | gatccGTTCTCCGAACGTGTCACGTAATTCAAGAGATTACGTGACACGTTCGGAGAATTTTTTc |
| shRNA NC Bottom strand | aattcAAAAAATTCTCCGAACGTGTCACGTAATCTCTTGAATTACGTGACACGTTCGGAGAACg |
| shRNA 1 Top strand | GatccGCAGGAAATGGCTGACTAGATTCAAGAGATCTAGTTCAGCCATTTCCTGCTTTTTTc |
| shRNA 1 Bottom strand | aattgAAAAAAGCAGGAAATGGCTGAACTAGATCTCTTGAATCTAGTTCAGCCATTTCCTGCg |
| BECN1-homo-170 sense（5'-3'） | GGUCUAAGACGUCCAACAATT |
| BECN1-homo-170 antisense（5'-3'） | UUGUUGGACGUCUUAGACCTT |
| ATG5-homo-2514 sense（5'-3'） | GCAUACAAUCUCAGAAACUTT |
| ATG5-homo-2514 antisense（5'-3'） | AGUUUCUGAGAUUGUAUGCTT |
