# Supplementary material for: BRCA1 affects the resistance and stemness of SKOV3‐derived ovarian cancer stem cells by regulating autophagy
Source: Cancer Med. 2019 Jan 12;8(2):656–68. doi: 10.1002/cam4.1975 (PMC6382722; doi:10.1002/cam4.1975)
Supplement: Supplementary file 5 [file CAM4-8-656-s005.docx]

| Table S2. The names, article numbers and manufactures  of the antibodies used in this study | | |
| --- | --- | --- |
| Antibody | Item No. | Manufacturer |
| mouse monoclonal anti-BRCA1 | NBP1-41185 | Novus, USA |
| rabbit monoclonal anti-Beclin1 | ab207612 | Abcam, USA |
| rabbit monoclonal anti-P Glycoprotein | ab168837 | Abcam, USA |
| rabbit monoclonal anti-SQSTM1/p62 | #8025 | Cell Signaling Technology, USA |
| rabbit monoclonal anti-LC3A/B | #12741 | Cell Signaling Technology, USA |
| rabbit monoclonal anti-Atg7 | #8558 | Cell Signaling Technology, USA |
| rabbit monoclonal anti-Atg5 | #12994 | Cell Signaling Technology, USA |
| mouse monoclonal anti-GSTP1 | #3369 | Cell Signaling Technology, USA |
| rabbit monoclonal anti-53BP1 | ab175188 | Abcam, USA |
| mouse monoclonal anti-Bcl-2 | #15071 | Cell Signaling Technology, USA |
| rabbit monoclonal anti-Caspase-3 | #9662 | Cell Signaling Technology, USA |
| rabbit monoclonal anti-ABCG2 | #42078 | Cell Signaling Technology, USA |
| rabbit monoclonal anti-Nanog | #4903 | Cell Signaling Technology, USA |
| rabbit polyclonal anti-Oct-4 | ab18976 | Abcam, USA |
| GAPDH | KC-5G4 | Aksomics, Shanghai, China |
