# Supplementary material for: BRCA1 affects the resistance and stemness of SKOV3‐derived ovarian cancer stem cells by regulating autophagy
Source: Cancer Med. 2019 Jan 12;8(2):656–68. doi: 10.1002/cam4.1975 (PMC6382722; doi:10.1002/cam4.1975)
Supplement: Supplementary file 6 [file CAM4-8-656-s006.docx]

| Table S3. The primers used in this study | | |
| --- | --- | --- |
| Target | Positive-sense strand | Antisense strand |
| GAPDH | AGGTGAAGGTCGGAGTCA | GGTCATTGATGGCAACAA |
| BRCA1 | GGCTATCCTCTCAGAGTGACATTT | GCTTTATCAGGTTATGTTGCATGG |
| NANOG | ATTTGCGGCCGCATGAGTGTGGGTCTTC | CGGGATCCTCATATTTCACCTGGTGGAG |
| POU5F1 | CTGAGGTGCCTGCCCTTCTA | CCAACCAGTTGCCCCAAAC |
| ABCG2 | GGGTTCTCTTCTTCCTGACGACC | TGGTTGTGAGATTGACCAACAGACC |
| CD44 | TGAGCATCGGATTTGAGA | CATACTGGGAGGTGTTGGA |
| BECN1 | ACCAGATGCGTTATGCCC | CAGCCTGAAGTTATTGATTGTG |
| ATG7 | GTTGTTTGCTTCCGTGAC | TGCCTCCTTTCTGGTTCT |
| ATG5 | CCATCAATCGGAAACTCA | AGCCACAGGACGAAACAG |
| ABCB1 | TACCTCCAGTTTCCTTTT | TTCTGTCGTTTTGTTTCA |
