# Supplementary material for: BRCA1 affects the resistance and stemness of SKOV3‐derived ovarian cancer stem cells by regulating autophagy
Source: Cancer Med. 2019 Jan 12;8(2):656–68. doi: 10.1002/cam4.1975 (PMC6382722; doi:10.1002/cam4.1975)
Supplement: Supplementary file 7 [file CAM4-8-656-s007.docx]

| Table S4. Clinicopathologic characteristics of  ovarian serous adenocarcinoma | | |
| --- | --- | --- |
| Parameter | No. of cases (total=20) | % |
| Age (years) |  |  |
| ＜50 | 9 | 0.45 |
| ≥50 | 11 | 0.55 |
| Surgery staging |  |  |
| I~II | 2 | 0.10 |
| III~IV | 18 | 0.90 |
| Histological grade |  |  |
| Moderately-High | 4 | 0.20 |
| Poorly | 16 | 0.80 |
| Lymph node metastasis |  |  |
| Detected | 17 | 0.85 |
| Not detected | 3 | 0.15 |
